# Supplementary material for: Bayesian refinement of protein structures and ensembles against SAXS data using molecular dynamics
Source: PLoS Comput Biol. 2017 Oct 18;13(10):e1005800. doi: 10.1371/journal.pcbi.1005800 (PMC5662244; doi:10.1371/journal.pcbi.1005800)
Supplement: S1 Archive — (BZ2) [file pcbi.1005800.s011.tar.bz2 › gromacs_4.62_bayesian_saxs_refi/share/html/online/g_nmens.html]

g\_nmens


| g\_nmens Main Table of Contents |

**VERSION 4.6  
Sat 19 Jan 2013**

---

### Description

g\_nmens generates an ensemble around an average structure
in a subspace that is defined by a set of normal modes (eigenvectors).
The eigenvectors are assumed to be mass-weighted.
The position along each eigenvector is randomly taken from a Gaussian
distribution with variance kT/eigenvalue.

By default the starting eigenvector is set to 7, since the first six
normal modes are the translational and rotational degrees of freedom.

### Files

| option | filename | type | description |
| --- | --- | --- | --- |
| **-v** | eigenvec.trr | Input | Full precision trajectory: trr trj cpt |
| **-e** | eigenval.xvg | Input | xvgr/xmgr file |
| **-s** | topol.tpr | Input | Structure+mass(db): tpr tpb tpa gro g96 pdb |
| **-n** | index.ndx | Input, Opt. | Index file |
| **-o** | ensemble.xtc | Output | Trajectory: xtc trr trj gro g96 pdb |

### Other options

| option | type | default | description |
| --- | --- | --- | --- |
| **-[no]h** | bool | no | Print help info and quit || **-[no]version** | bool | no | Print version info and quit || **-nice** | int | 19 | Set the nicelevel || **-xvg** | enum | xmgrace | xvg plot formatting: xmgrace, xmgr or none || **-temp** | real | 300 | Temperature in Kelvin || **-seed** | int | -1 | Random seed, -1 generates a seed from time and pid || **-num** | int | 100 | Number of structures to generate || **-first** | int | 7 | First eigenvector to use (-1 is select) || **-last** | int | -1 | Last eigenvector to use (-1 is till the last) |

---

http://www.gromacs.org  
gromacs@gromacs.org
